# My fear, my morals: a surgeon’s perspective of the COVID crisis

**DOI:** 10.1186/s13010-020-00094-3

**Published:** 2020-10-14

**Authors:** Shabir A Dhar, Zaid A Wani

**Affiliations:** 1 SKIMS MC Bemina, Srinagar, Kashmir 190020 India; 2 GMC Srinagar, Srinagar, Kashmir 190020 India

As the Corona Virus pandemic expands its footprint, the health care worker continues to man the frontlines of this, seemingly, relentless battle. Initially the surgical specialties had a marginal role in the patient care, but as the timeline has expanded, the surgeon is being increasingly called upon to participate in this prolonged tussle [1].

A surgeon has a rather privileged position in the society. By the very dint of his skills, perhaps this privilege extends to the implied hierarchies within the medical profession too. It is a huge honor and an equally big responsibility to have a patient entrust his body to someone. A trust that allows a surgeon to explore the otherwise unreachable sanctums of the human body through approaches that do not exist naturally. No matter how great a surgeon’s skill, the patient’s faith will always outweigh the skill on the moral scale. A faith that should to be respected and treated with utmost care. A surgeon does need to feel humbled and thankful for the relatively unique relationship he shares with his patient.

In most mass casualty incidents [MCI], the surgeon is often in the middle of the fight put up by communities and the healthcare systems. That is until the coronavirus visited us. Never in the recent memory has the surgeon felt more ill-equipped and never has the surgical skill set been so unimportant in the scheme of things.

It is quite understandable that the medical community was contemptuous and envious of us, in equal measure, during the initial days of the Pandemic. Contemptuous because of our relative uselessness to the task of confronting the virus and envious because of the safety it entailed. We also felt a similar set of emotions at a more variable and profound level. It was distressing to see our colleagues walk the hallways with their cheeks and noses having mask marks alluding to long hours of duty. They were the real heroes and heroines. We, meanwhile, were part of staff rationing and our work was to follow protocol by staying within our rooms and offices. At best we could send message morale boosters via phone. Within the walls of our office we reassessed the famous line from Charles Dickens ‘No one is use less in this world who lightens the burden of it to anyone else’. All this while, ethical debates continued to occur in our minds. How could we practice beneficence, non-maleficence, autonomy and justice from our uniquely ineffective roles [2, 3]?

Questions about the pandemic enforced surgical prioritization and the potential problems that the patient might face due to the delay, down the line, continue to challenge our ethical principles. Good judgement in the face of uncertainty is required more than ever. Whilst preservation of the work force is a vital component of the COVID management policy, but altruism does make some of us ask for redeployment in non-surgical roles. But this altruism is a potential source of interference in the normal hierarchy on the round. A senior surgical consultant, working as a resident, can be quite disrupting on a medical round.

The COVID confronts the doctor in three broad patient groups. The asymptomatic patient, the patient in the ICU and the patient needing surgery in the operation theatre. There is consensus that the healthcare worker most at risk is the one in the operation theatre. Both the anesthetist and the surgeon independently generate aerosols while operating in a relatively cloistered environment creating an ideal atmosphere for transmission.

As the number and range of COVID patients needing surgical intervention grows, me and my surgical colleagues are increasingly realizing that we have to join the battle at the deep end, first up. Throughout our lives we train to perform in the operation theatre as dispassionately as we can. While the surgeon’s overall personal risk is lower due less work during the pandemic, but whatever work that they do is inherently riskier due to close contact with potentially infected body fluids and parts. A stable mind, heart and hand is on the ultimate wish list of every surgeon. Then, you may ask, how is surgery during the COVID pandemic any different. The answer lies in, trying to explain, the maelstrom unleashed by the clash of fear and moral uprightness in our hearts by the virus.

The dominant view in ethics literature is that no individual health professional has a specific positive obligation to treat a patient when doing so places the professional at risk. Literature also clearly mentions that doctors have a strong duty to treat, therefore any proposed means of allocating staff that maximizes benefit to the patient might be justified [4]. The staff allocation varies across the spectrum of offering no choice to the staff, giving them individual choice or offering collective choice. According to Malon et al. in the face of personal risk, the obligation to treat position does not outweigh the duties professionals have to themselves and their families [5].

A common problem, that is not restricted to the surgeon, is the fear that we may be taking the virus home. It is a terrifying choice between the obligation of offering optimum patient care on one side and the small but definite likelihood of infecting our old parents and young children. No one can make this choice with any degree of logic and no one should have to. It is morbidly akin to that terrifying choice that the Joker offers to the people of Gotham. My parent or my patient? It probably is not that stark but is realistic enough to nag the mental health of every doctor in this fray.

As the authorities develop and change their policies especially regarding the management of COVID positive pregnant patients, our obstetric surgeons feel as if they are riding an emotional roller coaster. One moment they hear, thou shalt and the next thou shalt not! Even as one department is designated entirely for operating only COVID positive patients, we wonder how long their forbearance will last. As their risk multiplies they must also grapple, at a far more profound level, with a very disturbing question. Me, my parent or my patient? I have no idea how one can counsel, help or partly shoulder this crushing burden that they must be discussing, every day, in their minds.

The Hippocratic oaths, original and modern, do not hold an answer to this distressing and unusual choice. We are taught to choose good over evil and we feel quite fulfilled whenever we pick the correct side in a clear-cut binary, but this is a situation where the hardest thing and the right thing are confusingly interwoven. As responsible practitioners of the science of healthcare, there is a fine balance between sonhood, fatherhood, brotherhood and our important moral responsibilities as doctors. In many ways duties to one’s family are also a part of medical science albeit in the domain of psychology. We learn, listen, teach and train on the dinner table using our insight into the human brain to create reasonable arguments and discussions. Amidst these moments of care and intimacy, uncomfortable, distressing and nagging thoughts arise about being potential infection vectors ourselves.

Corona duties also bring us face to face with patients. Their loneliness and struggle against the disease and its protocols is very visible. As they see us in our white dispassionate white PPE suits, a distinct lack of connection pervades the air. The facelessness of the healthcare worker is a sort of condemnation. Reaching out across this safety barrier is very important, but protocol prohibits doffing the barrier. The only silver lining on these rounds is the likelihood that amidst the isolation, perhaps a white clad figure is better than no figure at all. Over, a period of time, all doctors know that conscience is the ultimate guiding light and allows us to get over many difficult situations. But for once conscience is like a cube and all sides seem to be the same.

At the other end of the same spectrum is the referring surgeon. They are following protocol by referring these patients as per guidelines. But they must ask themselves a question. Are we, at a primeval level, giving into that basic instinct of self-preservation? Did we refer out of fear? Are our colleagues, who are receiving these referrals, at less risk than us? Are they better equipped than us? Does the poor patient benefit? These are morally disturbing questions, but I am sure they are occupying several minds and causing conflicting introspection. There is absolutely no room for finger wagging.

The pandemic has forced us surgeons to think of one group of people who we generally take for granted. The members of our operating team. Is it possible for us to minimize their exposure whilst ensuring patient safety? This is, perhaps, a situation where safety and solace do not lie in numbers. An infected resident or an infected paramedic consequent to the surgery places the surgeon, who is the team leader, in an unenviable position. Moral responsibility clashes with fear again. The choice is not easy [6]. Kramer et al. allude to virtue-based theories which justify practice in spite of personal risk. They also mention that exposure approaching suicide is not good. Perhaps the way forward is to assess the risk individually while keeping beneficence in mind [7].

As we grapple our fears, our understanding of the non-surgical colleagues becomes more nuanced. They continue to fight the main war and our admiration is ever increasing. So is our deepening responsibility and the fear and worry for their well-being. How can we be of greater help to them without compromising the quality of the patient care? Productive team dynamics with preservation of moral agency is critical amidst the unfolding pandemic.

In the midst of this mental conflict lies the poor unwell patient. Unaware, that the pandemic might have subtly shifted the prism through which his sickness is seen. That fear and morals are clashing in the minds of his surgeon. That trust is pushing at responsibility. That the immovable is fighting the irresistible. That he, the caregiver, has no easy answers to this mental enigma.

I, fervently, hope that this uncertainty is not the long-lasting legacy of this pandemic.

## Data Availability

Not applicable.

## Abbreviation

PPE: Personal protective equipment
